# Vancomycin-resistant enterococci (VRE) screening and isolation in the general medicine ward: a cost-effectiveness analysis

**DOI:** 10.1186/s13756-019-0628-x

**Published:** 2019-10-29

**Authors:** Stephen Mac, Tiffany Fitzpatrick, Jennie Johnstone, Beate Sander

**Affiliations:** 1 Institute of Health Policy, Management and Evaluation, University of Toronto, 155 College Street, Suite 425, Toronto, ON M5T 3M6 Canada; 2 Toronto Health Economics and Technology Assessment (THETA) Collaborative, University Health Network, 200 Elizabeth Street, 10th Floor, Room 247, Toronto, ON M5G 2C4 Canada; 3 Dalla Lana School of Public Health, University of Toronto, 155 College Street, 6th Floor, Toronto, ON M5T 3M7 Canada; 4 Department of Laboratory Medicine and Pathobiology, University of Toronto, 1 King’s College Circle, Toronto, ON M5S 1A8 Canada; 5 Public Health Ontario, 480 University Avenue, Suite 300, Toronto, ON M5G 1V2 Canada; 6 ICES, G1 06, 2075 Bayview Avenue, Toronto, ON M4N 3M5 Canada

**Keywords:** Infection control, Vancomycin-resistant enterococci, VRE, Hospital-acquired infection, Antimicrobial resistance, Health economics, Cost-effectiveness analysis

## Abstract

**Background:**

Vancomycin-resistant enterococci (VRE) are a serious antimicrobial resistant threat in the healthcare setting. We assessed the cost-effectiveness of VRE screening and isolation for patients at high-risk for colonisation on a general medicine ward compared to no VRE screening and isolation from the healthcare payer perspective.

**Methods:**

We developed a microsimulation model using local data and VRE literature, to simulate a 20-bed general medicine ward at a tertiary-care hospital with up to 1000 admissions, approximating 1 year. Primary outcomes were accrued over the patient’s lifetime, discounted at 1.5%, and included expected health outcomes (VRE colonisations, VRE infections, VRE-related bacteremia, and deaths subsequent to VRE infection), quality-adjusted life years (QALYs), healthcare costs, and incremental cost-effectiveness ratio (ICER). Probabilistic sensitivity analysis (PSA) and scenario analyses were conducted to assess parameter uncertainty.

**Results:**

In our base-case analysis, VRE screening and isolation prevented six healthcare-associated VRE colonisations per 1000 admissions (6/1000), 0.6/1000 VRE-related infections, 0.2/1000 VRE-related bacteremia, and 0.1/1000 deaths subsequent to VRE infection. VRE screening and isolation accrued 0.0142 incremental QALYs at an incremental cost of $112, affording an ICER of $7850 per QALY. VRE screening and isolation practice was more likely to be cost-effective (> 50%) at a cost-effectiveness threshold of $50,000/QALY. Stochasticity (randomness) had a significant impact on the cost-effectiveness.

**Conclusion:**

VRE screening and isolation can be cost-effective in majority of model simulations at commonly used cost-effectiveness thresholds, and is likely economically attractive in general medicine settings. Our findings strengthen the understanding of VRE prevention strategies and are of importance to hospital program planners and infection prevention and control.

## Introduction

Vancomycin-resistant enterococci (VRE) are a class of antimicrobial resistant (AMR) bacteria most commonly transmitted within healthcare settings [1]. While immunocompetent patients have a low risk of acquiring VRE infections post-colonisation, other patient groups (e.g. immunocompromised, oncology, transplant) are at a higher risk of developing VRE-related bacteremia and other infections [2]. Consequently, patients who develop VRE-related infections require longer hospital stays, have a higher risk of mortality, and substantially higher medical costs. A study from Canada estimated the mean attributable cost and length of stay for patients with VRE colonisation/infection to be $17,949 and 13.8 days, respectively, when compared to patients without VRE [3].

Guidelines for control of VRE from health agencies (e.g. Centers of Disease Control and Prevention) in the United States and the United Kingdom recommend control of VRE spread through vancomycin usage, screening and isolation of patients with VRE in hospital settings, education, cleaning and contact precautions (e.g. gloves) [4, 5]. Similarly in Canada, provincial committees recommend the implementation of active VRE screening programs for patients at high-risk of VRE colonisation [6]. Risk factors for VRE colonisation include: previous admission to healthcare facilities (e.g. hospital); dialysis recipient; transfer from long-term care facilities; and previous receipt of certain classes of antibiotics (e.g. cephalosporin) [6].

In 2014, the Canadian Agency for Drugs and Technology for Health (CADTH) conducted a rapid response review on the cost-effectiveness of patient screening and isolation for VRE and identified one economic evaluation from France, where the direct cost of an outbreak triggered by a failure in systematic VRE screening had a direct cost of €60,524 [7]. Two economic evaluations from hospital settings reported a net benefit of using a VRE control strategy [8, 9].

Based on the current literature, there are no cost-effectiveness analyses for VRE screening and isolation practices that included health outcomes in evaluating the value of this control strategy. The objective of our study was to conduct a cost-effectiveness analysis of active VRE screening and isolation compared to no VRE screening and isolation in the general medicine ward of a tertiary care hospital. Due to conflicting evidence on the value of prevention programs for VRE, we decided to model a general medicine ward instead of an intensive care unit (ICU) because of its heterogeneous nature (i.e. varying patient risk for VRE colonisation and infections). Evidence from this model can inform decision-makers, program planners and clinicians contemplating control strategies for healthcare-associated VRE-related infections.

## Methods

A cost-effectiveness analysis (CEA) was conducted from the Ontario healthcare payer perspective (Ministry of Health and Long-Term Care). Health outcomes were accrued over a patients’ lifetime and included: healthcare-associated VRE colonisations, VRE-related infections (e.g. bacteremia and other infections), deaths subsequent to VRE infection, and quality-adjusted life years (QALY). All publicly-funded healthcare costs (2017 Canadian dollars) were included. The primary outcomes were total healthcare costs, QALYs, and the incremental cost-effectiveness ratio (ICER) expressed in $ per QALY gained. Cost-effectiveness of VRE screening and isolation was assessed against the commonly used cost-effectiveness threshold (CET) of $50,000 per QALY gained [10]. We followed CADTH guidelines and reported outcomes discounted at 1.5% [11].

### Model structure and patient population

**Fig. 1:**
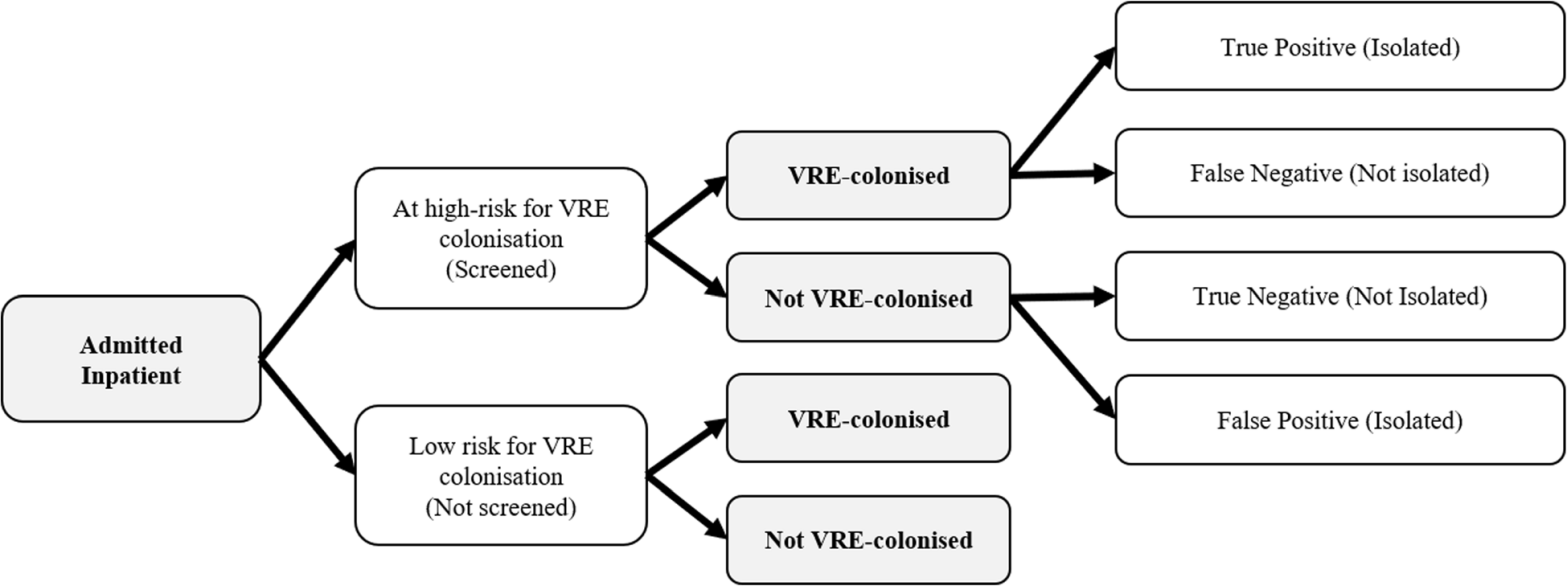
Schematic illustrating the possible trajectory of an admitted inpatient (screened or not, depending on the strategy)

**Fig. 2:**
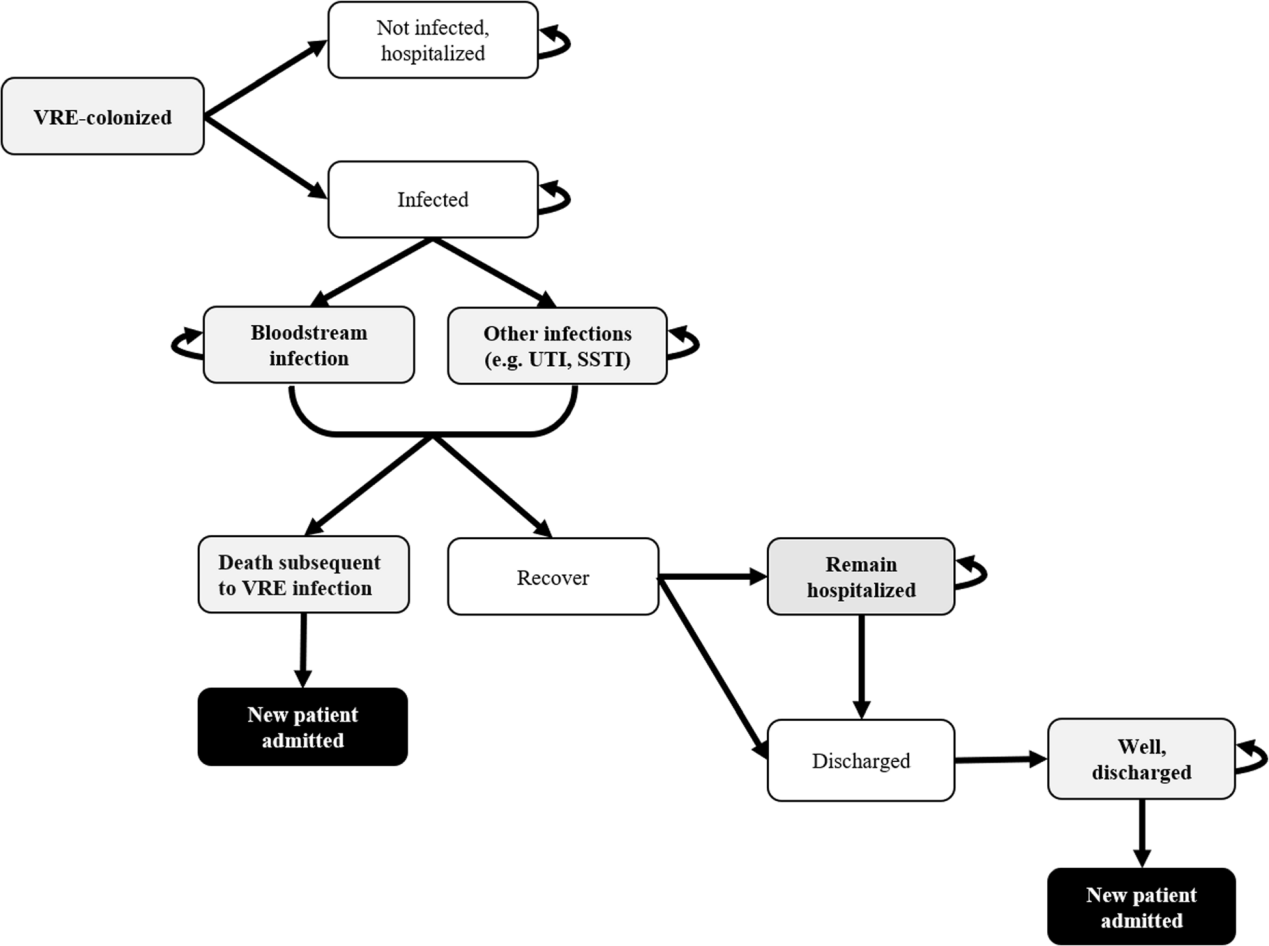
Schematic illustrating the trajectory of vancomycin-resistant enterococcus (VRE)-colonised patient

**Fig. 3:**
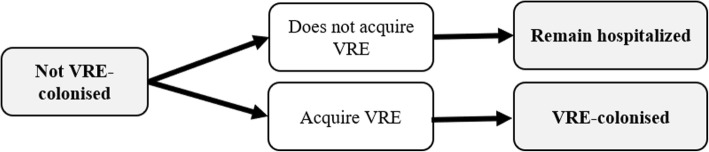
Schematic illustrating the possible trajectory of patient not VRE-colonised

A microsimulation model was developed to capture the natural history of VRE health burden starting at hospital admission. Schematics of the model are presented in Figs. 1, 2 and 3. The model simulated a dynamic population of 20 patients in the general medicine ward, i.e., patient flow was simulated by admitting a new patient to the ward once an existing patient was discharged back into the community, or died during their hospital stay. Admitted patients were considered to be from the community; we did not take into account entry from long-term care facilities, readmissions, or ICU step-downs. For base-case analysis, we evaluated the cost-effectiveness of VRE screening and isolation through 1000 admissions, approximating 1 year. After 1000 admissions, hospital admissions stopped, and patients were followed over their lifetime. All modelling and analyses were conducted using TreeAge Pro 2018 (TreeAge Software, Inc., Williamstown, MA).

### VRE transmission

A two-state dynamic transmission component simulated VRE transmission. The probability of acquiring VRE responds to changes to the number of VRE-colonised patients in the ward who are not isolated and was modeled using the following equation [12]:

$$ {\mathrm{C}}_{\mathrm{t}+1}/{\mathrm{S}}_{\mathrm{t}}=1\hbox{-} {e}^{\hbox{-} \beta Ct/N} $$

Where t represents the specific cycle or time period, C_t + 1_ is the number of patients who are VRE colonised (but not isolated) in the current cycle, N is total number of patients, S_t_ represents the total number of patients susceptible to VRE colonisation in the previous cycle, and *β* is the basic reproductive number of VRE. The basic reproductive number was defined as the number of new infections generated per infected (non-isolated) individual per unit of time. For our model, we assumed a constant basic reproductive number of 1.32.

### Key assumptions

Several key assumptions were made on VRE transmission and isolation parameters. These included: 1) VRE rectal swab screen are completed concurrently with Methicillin-resistant *Staphylococcus aureus* (MRSA) rectal swab screening (i.e., only additional cost is processing the swab), and results are delivered within 24 h, a period in which colonized patients can contribute to transmission; 2) transmission is based solely on mass-action mixing; 3) optimal adherence to isolation (i.e. isolation is 100% effective in reducing transmission); 4) cost of private (single-bed) room, which is typically considered hospital revenue, is captured in the healthcare payer perspective; 5) the general medicine ward has 20 single-bed rooms, always at maximum capacity; and 6) colonization status of the prior patient in the room was not factored into transmission.

### Data sources

A targeted literature search was conducted to extract outcome probabilities, costs and quality-of-life parameters related to VRE health states (Table 1). When possible, Canadian-specific parameters were used. Where “assumption” is indicated in Table 1, we were guided by expert opinion.

**Table 1:** Input parameter base-case values, plausible ranges and distributions

Variable	Base-case value	Range	Range Type	Standard Error	Distribution	Source
VRE-Related Parameters						
Beta, basic reproductive number	1.32	1.03–1.46	Full	0.12	Gamma	Satilmis 2016 [13]
VRE prevalence, general	0.023	0–0.18	Full	0.001	Beta	Williams 2015 [14]
VRE prevalence, high-risk patients	0.092	0–0.36	Plausible	0.002	Beta	Conly 2001 [15]
LOS | without VRE infection, days	3	1.0–6.0	Full (IQR)	0.38	Gamma	Johnstone 2018 [2]
LOS | other VRE infection, days	6	1.0–6.0	Full (IQR)	0.77	Gamma	Assumption; Johnstone 2018 [2]
LOS | VRE-bacteremia, days	39	22.0–81.0	Full (IQR)	4.97	Gamma	Johnstone 2018 [2]
Screening Parameters						
Sensitivity, rectal swab	0.991	0.95–1.00	Full	0.02	Beta	Stamper 2010 [16]
Specificity, rectal swab	0.949	0.92–0.97	Full	0.01	Beta	Stamper 2010 [16]
Effectiveness of isolation	1.00	0.75–1.00	Plausible	–		Assumption
Discount rate, annual	0.015	0–0.03	Full	–	–	CADTH 2017 [11]
Patient Parameters and Transition Probabilities						
Average age high-risk, years	61	–	–	1.15	Normal	Johnstone 2018 [2]
Probability infected | colonised	0.025	0.018–0.031	Plausible	0.003	Beta	Williams 2015 [14]
Probability bacteremia | infected	0.155	0.12–0.19	Plausible	0.02	Beta	Saunders 2004 [17]
Odds ratio bacteremia | infected, high-risk	1.55	0.56–4.29	Full	1.68	Lognormal	Johnstone 2018 [2]
Average days of treatment for BSI	14	11–18	Plausible	1.79	Gamma	Daneman 2016 [18]
Average days of treatment for other infections	7	5–9	Plausible	0.89	Gamma	Daneman 2016 [18]
Probability of death from VRE bacteremia, average, 14 days	0.37	0.27–0.46	Plausible	0.05	Beta	Billington 2014 [19]
Probability of death from VRE bacteremia, high-risk, 14 days	0.46	0.35–0.58	Plausible	0.06	Beta	Linden 1996 [20]
Number of room visits by all HCW, per day	24	18–30	Plausible	3.06	Normal	Assumption
Costs						
Rectal swab screen	3.13	2.35–3.91	Plausible	0.40	Gamma	Muto 2002 [9]
Culture, positive test	21.36	16.02–26.7	Plausible	2.72	Gamma	Muto 2002 [9]
Culture, negative test	8.97	6.73–11.21	Plausible	1.14	Gamma	Muto 2002 [9]
PPE, per room visit	2.10	1.58–2.63	Plausible	0.27	Gamma	Muto 2002 [9]
Nurse time, per test	7.12	5.34–8.9	Plausible	0.91	Gamma	Muto 2002 [9]
Private room, daily	290	245–410	Full	–	–	St. Joseph’s Hospital 2017 [21]
Antibiotics, bacteremia, daily	524.22	393.17–655.28	Plausible	66.87	Gamma	Nasr 2011 [22]
Antibiotics, other infections, daily	35.8	26.85–44.75	Plausible	4.57	Gamma	Nasr 2011 [22]
Utilities						
VRE bacteremia	0.56	0.51–0.61	Full	0.023	Beta	Lee 2010 [23]
Other local infections (UTI)	0.60	0.58–0.62	Full	0.01	Beta	Haran 2005 [24]
Inpatient	0.642	0.54–0.74	Full	0.05	Beta	Tengs, 2000 [25]; Selai 1995 [26]
Mild depression, no treatment	0.88	0.84–0.92	Full	0.02	Beta	Revicki 1997 [27]
Well, chronic conditions, recovered from previous VRE-related infection	0.86	0.34–0.89	Full	0.15	Beta	Mittmann 1999 [28]
Well, chronic conditions, no previous VRE-related infection	0.93	0.88–0.94	Full	0.083	Beta	Mittmann 1999 [28]

*BSI* bloodstream infection, *CADTH* Canadian Agency for Drugs and Technology in Health, *HCW* healthcare workers, *IQR* interquartile range, *LOS* length of stay, *PPE* personal protective equipment, *UTI* urinary tract infection, *VRE* vancomycin-resistant enterococcus

#### Probabilities

The basic reproductive rate for VRE was uncertain and can vary depending on the environment. We used results from a meta-analysis of 10 studies that reported a reproductive rate of 1.32 (95% CI, 1.03–1.46) [13]. Length of stay (LOS) estimates used for patients with VRE infections was 39 days (IQR, 22–81 days) and without VRE infections was 3 days (IQR, 1–6 days), extracted from a case-control study in Canada [2]. We used a screening rectal swab sensitivity of 0.99 (95% CI, 0.952–1.00) and specificity of 0.948 (95% CI, 0.922–0.968) from an United States study evaluating the swab detection of *E. faecium* and *E. faecalis* [16]. Prevalence of VRE for low-risk patients was 0.023, which was extracted from a Canadian study in 2012 [14]. The probability that a patient was at “high-risk” of colonisation was guided by the average age (61 years) of the cohort of patients who acquired VRE-bacteremia in Canada [2]. All-cause mortality from all-causes were derived from life tables from Statistics Canada [29].

#### Utilities

To properly value health outcomes for CEAs, we used health state utility values (utilities), which is a preference-based value expressing the quality-of-life associated with health states [30]. Utilities for this study could have ranged between 0 (health state equivalent to death) to one (perfect health). The utility of a VRE-colonised patient was considered to be the same as that of a general inpatient (0.642), which was obtained from a mixed population of inpatients using the EuroQol rating scale [25]. The utility for the well outpatient state was derived from a study of community-dwelling adults using the Health Utilities Index to be 0.93 (0.86 for patients who recovered from a VRE-related infection) [28]. Due to data limitations, bacteremia utility (0.56) was extracted from a MRSA-related bacteremia study [23]. Since urinary tract infections (UTI) represented the greatest percentage of VRE-related infections [3, 31], we used the UTI utility of 0.60 for all other infections [24]. We assumed a disutility with being isolated (i.e. being isolated leads to less visits from healthcare workers, reduced socialization, and space confinement), which was equivalent to mild depression (untreated), and applied a multiplicative 0.895 reduction factor [27].

#### Costs

All direct costs were extracted from the literature (Table 1). We counted the cost of the screening as a one-time upfront cost at ward admission between $12 and $24, depending on the culture result (positive results being more expensive due to additional microbiologist time required) [9]. All costs were converted and standardized to 2017 Canadian dollars. For private room costs, we used the median from estimates across Ontario ($290 per night) [21].

### Analysis

The base-case analysis was defined as follows: screening with 95% specificity and 99% sensitivity, VRE basic reproductive number of 1.32 [13], and mean age of high-risk patients at 61 years [2]. The baseline prevalence of VRE was 0.023 and we assumed patients at higher risk for VRE colonisation were four times more likely to be colonised (0.092). The base-case analysis was conducted from a Canadian perspective.

We conducted multiple scenario analysis including: universal screening and isolation for all patients, increased duration of the program (5000 admissions), number of beds, and a lower effectiveness (compliance) of the isolation program.

We conducted a probabilistic sensitivity analysis (PSA) using gamma distributions for costs, beta distributions for utilities and transitional probabilities, and normal distributions for other patient or VRE-related parameters (see Table 1). From the PSA, we generated a cost-effectiveness acceptability curve (CEAC) to determine the probability of VRE screening and isolation being cost-effective at CET of $0 to $100,000 per QALY. We also assessed expected value of perfect information at several CETs to assess the value of information; i.e., whether or not to invest more resources to reduce parameter uncertainty. As recommended by CADTH, we did not conduct deterministic sensitivity analysis because of model stochasticity and the non-linear relationship of VRE prevalence and transmission parameters. We reported results following the Consolidated Health Economic Evaluation Reporting Standards (CHEERS) Guidelines (Additional file 1) [32].

## Results

### Base-case analysis

In Table 2, we summarized the estimated health outcomes, costs and ICER for the VRE screening and isolation strategy compared to no VRE screening and isolation over 1000 admissions for our base-case analysis. We calculated the difference in the health outcomes and the relative change using the “no VRE screening and isolation” strategy as the baseline. VRE screening and isolation reduced healthcare-associated VRE colonisations by six per 1000 patients (2/1000 with screening and isolation vs. 8/1000 without, 73% reduction), VRE-related infections by 0.6 per 1000 patients (5.7/1000 with screening and isolation vs. 6.3/1000 without, 10%), VRE-related bacteremia by 0.2 per 1000 patients (2.5/1000 with screening and isolation vs. 2.7/1000 without, 7%) and deaths subsequent to VRE infection by 0.1 per 1000 (0.5/1000 with screening and isolation vs. 0.6/1000 without, 8%).

**Table 2:** Base-case results (health and economic outcomes)

Outcomes	VRE screening and isolation	No VRE screening and isolation	Difference^a^ (%)
Non-isolated cases	11/1000	60/1000	−49/1000 (82%)
Healthcare-associated VRE-colonisation	2/1000	8/1000	−6/1000 (73%)
Infected cases	5.7/1000	6.3/1000	−0.6/1000 (10%)
VRE-related bacteremia	2.6/1000	2.8/1000	−0.2/1000 (7%)
Other VRE infections (e.g. UTI)	3.2/1000	3.6/1000	−0.4/1000 (12%)
Deaths subsequent to VRE infection	0.5/1000	0.6/1000	−0.1/1000 (8%)
ICER ($/QALY)			7850
Total costs ($)	118.37	6.72	112
Total QALY gained	20.5607	20.5465	0.0142

^a^Difference for health outcomes were calculated by subtracting “no VRE screening and isolation strategy” outcomes from “VRE screening and isolation strategy” outcomes. Percentage change was calculated relative to “no VRE screening and isolation strategy” outcomes

*ICER* incremental cost-effectiveness ration, *QALY* quality-adjusted life years, *UTI* urinary tract infection, *VRE* vancomycin-resistant enterococci

The incremental cost and effect for VRE screening and isolation was $110 ($118.37 with screening and isolation vs. $6.72 without), and 0.0142 QALY gained (20.5607 QALY with screening and isolation vs. 20.5465 QALY without), respectively. The ICER for VRE screening and isolation was $7850 per QALY gained.

### Uncertainty: probabilistic sensitivity analysis

**Fig. 4:**
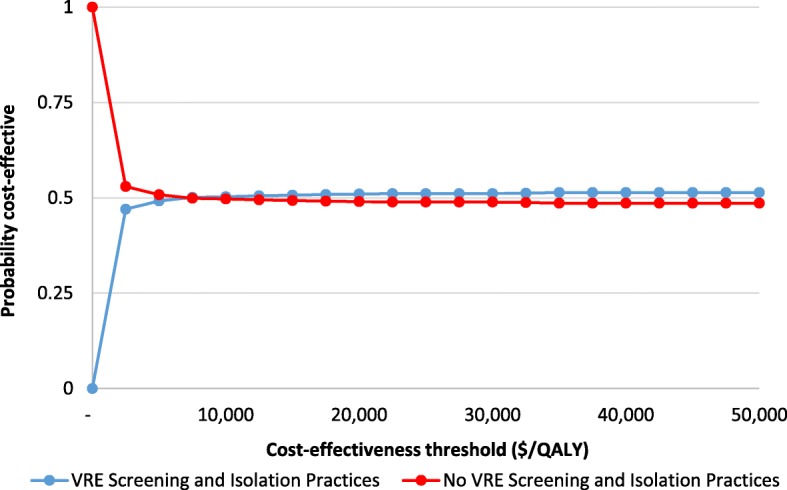
Cost-effectiveness acceptability curve (CEAC) for cost-effectiveness thresholds from $0 to $50,000/QALY

Figure 4 illustrates a CEAC where at low CETs below $7500/QALY, it was unlikely that VRE screening and isolation was a cost-effective strategy. At a CET of approximately $7500/QALY, VRE screening and isolation became more likely to be cost-effective (over 50% of the iterations). As the CET increased to $50,000 per QALY, the probability of this program being cost-effective asymptotes at approximately 51.4%.

Since VRE screening and isolation reached a plateau of 51% likelihood of being cost-effective, an expected value of perfect information (EVPI) analysis was conducted to determine the value of reducing further uncertainty at three points. At a CET of $7500, and $50,000 per QALY, the EVPI (assuming 1000 patients) was $1065, and $7093, respectively.

### Scenario analysis

In the scenario where the prevalence is lower (i.e. reduced by half; 0.0115), VRE screening and isolation becomes a dominated strategy: the program cost an additional $123 but resulted in fewer QALYs. On the other hand, we modeled a scenario similar to outbreaks in the literature where the VRE prevalence was about 10-fold higher (0.23), and estimated that VRE screening and isolation cost $122.79 for an incremental increase of 0.0525 QALY. Under this increased prevalence scenario over 1000 hospital admissions, the ICER was $2340/QALY. All scenarios are summarized in Table 3.

**Table 3:** Incremental cost-effectiveness ratios for VRE screening and isolation program in various scenarios

Scenario	Incremental Cost	Incremental QALYs	ICER ($/QALY)	Probability of CE (at $7500/QALY)	Probability of CE (at $50,000/QALY)
VRE Prevalence in-hospital, 10x (outbreak)	122.79	0.0525	2340	0.545	0.556^a^
Room costs excluded ($0)	20.58	0.0077	2682	0.506	0.508^a^
Number of beds in ward [33]	109.78	0.0093	11,812	0.505	0.518^a^
Program length (5000 admissions)	113.05	0.0023	50,094	0.457	0.499^a^
Isolation, decreased effectiveness (0.75)	99.52	0.0002	510,676	0.458	0.476^a^
Time horizon, 1 year	109.61	0.0001	856,297	0	0.259
Universal screening VRE screening and isolation	151.44	−0.0039	Dominated	0.484	0.500^a^
VRE Prevalence in-hospital, 0.5x	108.41	−0.0112	Dominated	0.479	0.501^a^

^a^Signifies asymptote at that probability at $50,000/QALY

*CE* cost-effectiveness, *ICER* incremental cost-effectiveness ratio, *QALY* quality-adjusted life year, *VRE* vancomycin-resistant enterococci

Scenario analysis was conducted where the private room costs were excluded due to conflicting views on whether these costs are considered from the Ontario healthcare payer perspective. In this scenario, VRE screening and isolation program cost an additional $20.58 for 0.0077 QALYs, resulting in an ICER of $2682/QALY. The number of beds in the simulated general medicine ward was increased to 30. The cost-effectiveness of VRE screening and isolation over 5000 admissions was also estimated. The estimated ICERs for these scenarios were $11,812/QALY and $50,094/QALY, respectively.

Universal VRE screening and isolation for all patients, regardless of whether they identified as high-risk for colonisation, was a dominated strategy (i.e. resulted in incremental cost of $151.44 and QALYs lost). We also estimated the cost-effectiveness of this program if the isolation effectiveness was reduced to 75%. In this scenario, VRE screening and isolation cost an additional $99.52 for 0.0002 QALYs, resulting in an ICER of $510,676/QALY.

## Discussion

Based on our base-case analysis, VRE screening and isolation for patients at high-risk for VRE colonisation prevented healthcare-associated colonisations, and ultimately VRE-related infections and deaths subsequent to infections. The program was considered cost-effective with an ICER of $7850 per QALY when compared to commonly used cost-effectiveness thresholds of $50,000/QALY [10].

Overall, our model’s results were consistent with the findings of several other published studies [34–37]. A study by Shadel et al. found that active VRE screening and isolation resulted in 91% of VRE colonisations being identified on an ICU; our model suggested 82% of VRE positive patients were isolated under an active, targeted screening strategy in a general medicine ward [34]. A mathematical model of a 10-bed ICU active screening program for VRE predicted 9.9 cases of VRE colonisation/infection prevented over 1000 model simulations in the ICU with a prevalence rate of 5% [35]. Similarly, our model predicts a reduction of 6 cases of VRE colonisations over 1000 admissions. Our model underestimated the effect of the VRE screening and isolation compared to both studies, likely because it was modeled after a general medicine ward which has a lower proportion of high-risk patients (for VRE colonisation and infection) than the ICU. However, similar to other studies, our model estimated that active VRE screening and isolation strategy was cost-effective by reducing the number of VRE-related bacteremia events by 2/10,000 patients [36, 37].

Our study has several limitations. Health state utilities were not specific to VRE infections and preference elicitation was heterogeneous. To address this and other parameter uncertainty (e.g. costs and transition probabilities), we conducted a PSA with the appropriate underlying distribution for all parameters to generate a CEAC for cost-effectiveness thresholds of $0 to $50,000/QALY. VRE screening and isolation was more likely to be cost-effective than no VRE screening and isolation at a CET of $7500/QALY or greater. However, as the CET increased to $50,000/QALY, the likelihood of the program being cost-effective in extended simulations remained steady at 51%, suggesting that stochasticity (randomness) is a significant factor in determining the value of this control program. This was expected for this type of intervention since individual level uncertainty with patients entering a general medicine ward and the baseline VRE prevalence can influence VRE transmission.

Our study assumed a general medicine ward that was set up with 20 single bed rooms, which may not be the configuration of all general medicine wards. In a scenario where 30-beds were used, the ICER increased to $11,812/QALY. These results suggest that an increase in the number of beds would still yield cost-effective VRE screening and isolation practices due to the homogeneous mixing assumption. This assumption was made despite knowing that VRE transmission can be highly complex and depend on colonization pressure and density of bacteria [33]. Incorporating such detail of VRE colonization levels within the transmission modeling of this CEA would require much more sophisticated VRE surveillance data that was not available. We did not explore the value of this program in which patients shared rooms. However, based on Hamel et al., the hazard ratio for VRE colonisation was 1.11 (95% CI, 1.02–1.21) for the number of roommate exposures per day [38]. Our estimates using a single-bed room assumption was a conservative approach, and therefore likely underestimated the cost-effectiveness of a VRE screening and isolation control program. Our model likely provided a conservative estimate of the cost-effectiveness (i.e. underestimates the value) of VRE screening and isolation due to key assumptions required for our analysis (e.g. did not incorporate time dependency within the ward, or re-admissions).

Isolation was assumed to be completely effective in our base-case analysis, which can be considered optimistic in current healthcare settings given the potential for human errors, and overall burden on healthcare workers [39, 40]. We performed a scenario analysis based on a study by Huskins and colleagues suggesting prevention effectiveness of 75% (range 62–82%) [40], and the ICER increased to $510,676/QALY. In this scenario, VRE screening and isolation would unlikely be cost-effective at commonly used thresholds. Due to stochasticity (randomness), it is likely that the cost-effectiveness and isolation effectiveness have a nonlinear relationship. This may be of note to decision-makers and infection prevention and control practitioners, to ensure implementation of this program is as seamless as possible.

Cost-effectiveness analyses for screening programs of other AMR bacteria such as carbapenemase-producing *Enterobacteriaceae* and MRSA have been published in the literature [41, 42]. Similar to these other economic evaluations on AMR bacteria screening and isolation, our results indicated that VRE screening and isolation was likely to be cost-effective. To our knowledge, this is the first cost-effectiveness analysis for VRE screening and isolation in any hospital setting that incorporated costs, health outcomes, and QALYs, accrued over a patient’s lifetime. We reported health outcomes per 1000 patients to allow for transferability of our results to general medicine wards in different jurisdictions. Moreover, the results of this cost-effectiveness analysis can be generalizable to other jurisdictions (countries) with similar healthcare system financing to Canada such as Australia, the United Kingdom, and parts of Europe. We also estimated the cost-effectiveness of this program in varying scenarios (e.g. varying VRE prevalence, number of beds) to provide decision-makers with economic evidence to support local health policy given the importance of local context.

Given the limited body of evidence in this area, we were unable to find a suitable source of data against which to validate our results. As more local research on AMR bacteria continues, it will allow for future models to be cross-validated to health outcomes using health administrative data, ward caseload (e.g. bed capacity), admission data (e.g. population characteristics), and number of VRE-related bacteremia cases.

## Conclusion

VRE screening and isolation for patients at risk for colonisation in the general medicine ward can be considered a cost-effective infection prevention and control intervention in this simulation study. The intervention’s cost-effectiveness varied depending on VRE prevalence and isolation effectiveness. This model would need to be adapted to more accurately estimate the impact in specific local contexts but can provide broad economic evidence to inform infection prevention and control practitioners, program planners and health policy decision-makers.

## Supplementary information

**Additional file 1.** CHEERS Checklist.

## Data Availability

All data generated or analysed during this study are included in this article.

## Abbreviations

AMR: Antimicrobial resistant
CADTH: Canadian Agency for Drugs and Technology for Health
CEAC: Cost-effectiveness acceptability curve
CET: Cost-effectiveness threshold
CHEERS: Consolidated Health Economic Evaluation Reporting Standards
CI: Confidence interval
EVPI: Expected value of perfect information
ICER: Incremental cost-effectiveness ratio
ICU: Intensive care unit
IQR: Interquartile range
LOS: Length of stay
MRSA: Methicillin-resistant *Staphylococcus aureus*
PPE: Personal protective equipment
PSA: Probabilistic sensitivity analysis
QALY: Quality-adjusted life year
UTI: Urinary tract infection
VRE: Vancomycin-resistant *enterococci*
